# To Remove or Not to Remove? The Challenge of Extracting the Template to Make the Cavities Available in Molecularly Imprinted Polymers (MIPs)

**DOI:** 10.3390/ijms12074327

**Published:** 2011-07-05

**Authors:** Rosa A. Lorenzo, Antonia M. Carro, Carmen Alvarez-Lorenzo, Angel Concheiro

**Affiliations:** 1 Department Química Analítica, Nutrición y Bromatología, Facultad de Química, Universidad de Santiago de Compostela, Avda. de las Ciencias, s/n, 15782-Santiago de Compostela, Spain; E-Mails: rosaantonia.lorenzo@usc.es (R.A.L.); tuchi.carro@usc.es (A.M.C.); 2 Department Farmacia y Tecnología Farmacéutica, Facultad de Farmacia, Universidad de Santiago de Compostela, 15782-Santiago de Compostela, Spain; E-Mail: carmen.alvarez.lorenzo@usc.es

**Keywords:** molecularly imprinted polymer (MIP), template removal, solvent extraction, physically-assisted extraction, ultrasound-assisted extraction, microwave-assisted extraction, pressurized-liquid extraction, subcritical water extraction, supercritical fluids extraction

## Abstract

Template removal is a critical step in the preparation of most molecularly imprinted polymers (MIPs). The polymer network itself and the affinity of the imprinted cavities for the template make its removal hard. If there are remaining template molecules in the MIPs, less cavities will be available for rebinding, which decreases efficiency. Furthermore, if template bleeding occurs during analytical applications, errors will arise. Despite the relevance to the MIPs performance, template removal has received scarce attention and is currently the least cost-effective step of the MIP development. Attempts to reach complete template removal may involve the use of too drastic conditions in conventional extraction techniques, resulting in the damage or the collapse of the imprinted cavities. Advances in the extraction techniques in the last decade may provide optimized tools. The aim of this review is to analyze the available data on the efficiency of diverse extraction techniques for template removal, paying attention not only to the removal yield but also to MIPs performance. Such an analysis is expected to be useful for opening a way to rational approaches for template removal (minimizing the costs of solvents and time) instead of the current trial-and-error methods.

## 1. Introduction

Molecular imprinting technology has undergone an enormous development in the last decade, being applied not only in the analytical field, where it was conceived, but also for environmental remediation, biotechnological processes, or development of vanguard drug delivery systems, among others [1–4]. Most advances have focused on the improvement of the polymerization techniques in order to achieve a precise control of the monomers arrangement and, thus, of the structure of the imprinted polymers (MIPs) at both the nanoscale level to render well-formed and stable imprinted cavities with selective features, and the macroscopic level to obtain materials of various formats, with different physical and chemical properties [5–7]. Versatile and reproducible synthesis approaches, based on accumulated experience from trial-and-error experiments and more recently on the application of rational design methodologies, enable facing up to an already high demand of applications and to explore new fields [8,9]. Nevertheless, although the polymerization step is a critical first phase, the removal of the template is without doubt a decisive maneuver for the optimal performance of the MIP [10]. Compared to the ample information available for optimizing the polymerization conditions regarding materials, energy and time costs, the removal step is the sister Cinderella. Few papers indicate the yield of removal after applying a certain technique [11,12] and even less explain the basis behind the use of a given extraction technique (a nice comparative study dates back 10 years ago [10]). Such a lack of attention has resulted in the removal being the least cost-effective step of the MIP development.

**Figure 1:**
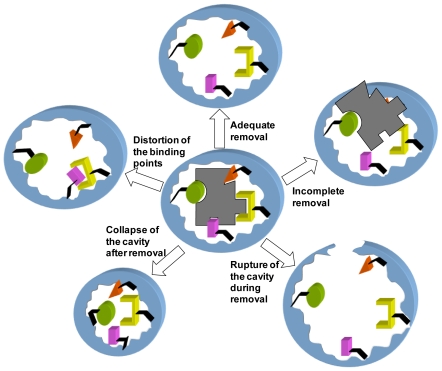
Scheme of the changes induced in the MIPs during the removal of the template.

Discontinuous immersion (incubation) in organic solvents or salt solutions and continuous extraction in a Soxhlet apparatus are the most traditional extraction techniques. Unfortunately, 100% removal of the template is hard to achieve even after exhaustive washing cycles, mainly due to poor accessibility of the solvent to highly cross-linked regions or to insufficient solubility of the template in the solvent to break the interactions with the imprinted cavity [10,13]. In fact, in the case of large molecules and proteins, permanence of nearly 25% of the original template has been reported [14,15]. That causes not only a decrease in the number of cavities suitable for rebinding (Figure 1), but also a bleeding of the residual template during elution of MIP-based chromatography columns for solid phase extraction [16] or during analysis [17]. Even in the case of MIPs prepared according to the self-assembly or non-covalent approach, the binding of the template to the components of the imprinted cavity can be so strong that extraction under drastic conditions is required [18]. Extreme pH or temperature applied for a long time can lead to the distortion and even rupture of the cavity during removal (Figure 1), resulting in MIPs of poor selectivity and recovery [10]. Furthermore, changes in the degree of swelling of the MIP network during extraction and subsequent desiccation can result in the collapse of the cavity, sterically hindering the entrance of target molecule, or in a distortion of the binding points or the strength of the interactions (Figure 1) [19,20].

**Figure 2:**
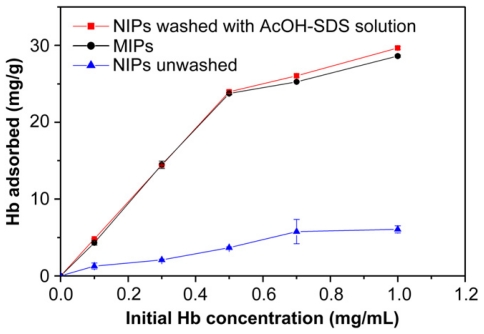
Hemoglobin (Hb) sorption isotherms on imprinted (MIP) and non-imprinted (NIP) polyacrylamide/chitosan semi-interpenetrated networks. The MIPs were washed with a 10% (v/v) AcOH solution containing 10% (w/v) sodium dodecylsulfate (SDS) to remove the template molecules, and then equilibrated in phosphate buffer pH 6.8. The non-imprinted network (NIPs) were either not washed or washed. Reproduced from Fu *et al.* [20] with permission from Elsevier.

It is interesting to note that for an adequate comparison of the MIP with the corresponding non-imprinted network (NIP), the NIP should not skip the removal step. Differences in the washing procedure have been shown to lead to unrealistically high selectivity factors of the MIPs, particularly in the case of swellable networks, due to promoted non-specific binding (Figure 2) [20].

To partially overcome these problems, some authors have proposed the synthesis in the presence of an analogue molecule instead of the template [21–23] or the use of covalently linked bi- or multi-functional monomers in order to completely avoid the presence of the template [24,25]. However, both approaches are quite limited since adequate analogue molecules are commonly quite hard to identify and the design of monomers that can render suitable functional groups after the rupture of a labile bond is not an easy task either. Therefore, the use of template molecules and the application of a removal step are still mandatory in most cases. As an exception, certain ion-selective electrodes do not require the removal of the template from the conducting polymer membrane [26].

**Figure 3:**
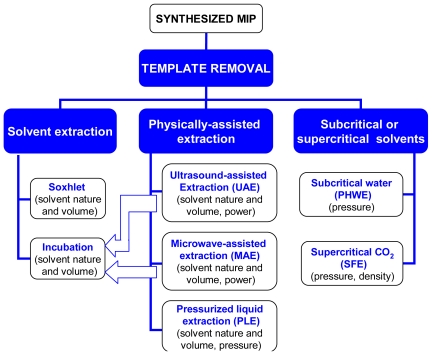
The three main approaches available for template removal: extraction with common solvents, physically-assisted solvent extraction, and extraction with subcritical or supercritical fluids. The variables that determine the yield of each process, in addition to temperature and time, and the interrelations among the techniques are also shown.

The current state-of-the-art on extraction comprises a wide variety techniques [27–29], some of them already well implanted in analytical chemistry but still scarcely applied for template removal. Each extraction technique has its own merits and to establish a decision tree for their selection is not easy since the nature and the stability of both the template and the MIP should be considered. Nevertheless, the following general rules should be taken into account: Simplicity of use, short operation time, environmentally friendly solvents, minimum amount of solvent, low economic cost, and possibility of becoming used at the industrial scale [30]. Starting from the extraction with common solvents, three main extraction strategies have been developed following green-chemistry and cost-effective principles: Solventless extraction, extraction using subcritical (e.g., pressurized hot water) or supercritical (e.g., CO_2_) solvents, and physically-assisted solvent extraction (*i.e*., extraction helped by the concomitant application of physical phenomena) [31]. Examples of the application of the last two approaches to the template removal are already available. The aim of this review is to offer an overall analysis of techniques suitable to remove the template, ranging from the most classical to emerging technologies, which is summarized in the diagram in Figure 3. The text is not intended to exhaustively describe each extraction technique, which can be found in general texts to which the reader is referred in each section, but to provide information about the advantages/limitations of each technique and the appropriateness for template removal based on the information available not only on removal yield but also on the performance of the obtained MIPs. A better knowledge of feasible template removal strategies is expected to give an extra push to the development of more efficient MIPs.

## 2. Extraction with Common Solvents

### 2.1. Conventional Soxhlet Extraction

Extraction with organic solvents using a Soxhlet apparatus is a standard method since its apparition nearly 135 years ago [32] and it is commonly used as the reference to compare the performance of other methods. Most EPA (US Environmental Protection Agency) and FDA (Food and Drugs Administration) official methods involve the use of Soxhlet apparatus for continuous extraction of analytes from solid matrices. Briefly, this technique consists in placing finely crushed MIP particles into a porous cartridge inside the extractor chamber. The extracting solvent (usually an organic solvent containing acid or base additives) is poured into a flask connected to the lower end of the extractor chamber. The solvent is heated, becomes volatilized and goes up into the extractor chamber. The condensed vapor drops into the cartridge entering into contact with the MIP particles and removing the template. When a certain level of liquid is reached, the solvent with the dissolved template goes down through a siphon to the flask. The process is continuously repeated for several hours. Since the solvent is re-circulated through the MIP particles, the process can be considered as a continuous extraction. Optimization of the template removal yield requires choosing the most adequate volatile solvent that solubilizes the template, the tuning of the solvent volume and the operation time.

The main advantages of Soxhlet extraction can be summarized as follows [33]: (i) the MIP particles (1–100 g) are repeatedly washed with fresh portions of the extracting solvent; (ii) the extraction is carried out with a hot solvent, which may favor the solubilization of the template; (iii) no filtration is needed after the extraction to collect the MIP particles; (iv) the equipment is affordable and the operator can be easily trained; and (v) versatility for being applied to almost any polymer matrix.

On the other hand, several disadvantages can also be mentioned [33]: (i) long extraction time is required (6–24 h); (ii) the amount of organic solvent used is quite large (50–300 mL), which may imply environmental concerns as well as a subsequent evaporation for quantifying the amount of template removed; (iii) risk of temperature-induced degradation of labile templates; (iv) the MIP particles remain mostly static during the process, which hinders the flow of the solvent around them and delays the extraction process; and (v) automation is difficult.

**Figure 4:**
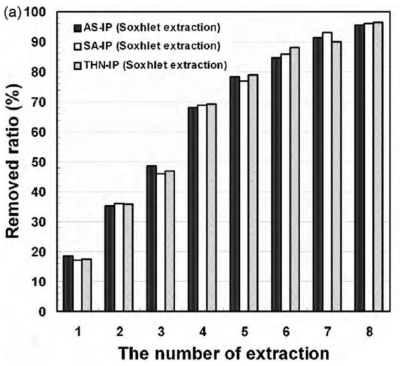
Removal ratio of the template from MIP particles (212 μm) as a function of the number of 8 h-cycles of Soxhlet extraction with ethanol. Reprinted from Byun *et al.* [39] with permission from Elsevier.

Literature about template removal using Soxhlet extraction is quite large since this technique has been applied to a wide variety of MIPs [34–38]. However, the number of papers that reports on the monitoring of the template removal is much less. For example, theophylline-imprinted polymers (2 g) prepared by using methacrylic acid (MAA) solely or combined with 2-hydroxyethyl methacrylate (HEMA) or acrylamide were extracted with 120 mL of methanol containing 10% acetic acid for 24 h. The amount of the extracted template, which was monitored at 271 nm, resulted in between 67 and 88% depending on the conomomers [38]. Dopamine-imprinted particles (439 mg) after being placed for continuous extraction in a Soxhlet apparatus (30 cycles, 80 mL, methanol:water, 1:1 v/v) still exhibited remarkably high levels of dopamine bleeding when subjected to elution with 0.05 M aqueous ammonium formate (pH 3): Methanol (3:1 v/v) solvent [11]. The bleeding was significantly larger than with that obtained with other extraction techniques, such as microwave-assisted extraction (MAE). Byun *et al.* [39] prepared MIPs for aspirin (AS), salicylic acid (SA) or 1,2,3,4-tetrahydro-1-naphthol (THN) with styrene, 4-vinylpyridine (4-VPy) as a functional monomer and divinylbenzene as the cross-linker. The complete removal of the templates using Soxhlet extraction with ethanol required eight cycles of 12 h, *i.e.*, 96 h in total (Figure 4) [39]. The removal profile indicates a linear increase in the amount of template removed during the first five cycles, followed by a decreased slope in the subsequent cycles.

### 2.2. Incubation with Solvents

Immersion of MIPs in solvents that can induce the swelling of the network and, at the same time, favor the dissolution of the drug is the simplest way to extract the template. The method is usually carried out under mild conditions and the chemical stability of the networks is not compromised, as may occur during Soxhlet extraction [40]. A high volume of medium (needed to create a high gradient concentration between the MIP and the solvent at the bulk), heating and stirring of the solvent medium or oscillation of the whole system can speed up the process, although in general the removal still requires several hours. For example, timolol-imprinted HEMA disks (0.7 mm thickness) prepared with methacrylic acid (MAA) or methyl methacrylate (MMA) as functional monomers lost 30% of the template when immersed in boiling water for 3 min. Nevertheless, the MIPs required nearly 24 h more for the removal of the 70% remanent timolol when placed in unstirred 0.9% NaCl aqueous solution, despite of being swelled in that medium [41]. Longer extraction times were required for 100% removal of norfloxacin or theophylline from similarly designed MIPs; e.g., immersion in boiling water for 15 min and then in NaCl 10 mM for 1 week, replacing the medium each 12 h, then in HCl 10 mM for 1 day and in water for 1 day more [42,43]. Experiments carried out to optimize the conditions to remove tobacco mosaic virus from virus-imprinted poly(allylamine) hydrogels cross-linked with ethylene glycol diglycidyl ether revealed that after five wash cycles in 1 M NaOH, each cycle lasting 6 h, 81.50% virus removal was achieved. The yield was only 0.12, 0.22 and 21.16 in water, 1 M NaCl, and 6 M urea, respectively [44,45]. Eight wash cycles with 1 M NaOH ensured more than 90% removal. In this medium the polymer can swell, the amine groups of the polymer become uncharged and the binding to the virus template is disrupted. Furthermore, the virus is unstable and disassembles [44].

The synthesis approach for preparing estradiol-MIPs has been shown to affect the template extraction [46]. MIPs prepared applying precipitation polymerization were washed with methanol/acetic acid (90:10 v/v; 3 × 10 mL), followed by acetone (1 × 10 mL). However, MIPs obtained by bulk polymerization and finely crushed required seven rounds of 15 mL methanol/acetic acid (4:1) followed by three rounds of acetonitrile (incubating for 1 h at 60 °C under agitation). Since the particle size was similar for both MIP types (20 μm), the authors justified the application of different removal protocols by the fact that bulk polymerization gives particles with deeper cavities compared to those resulted from precipitation polymerization [46].

**Figure 5:**
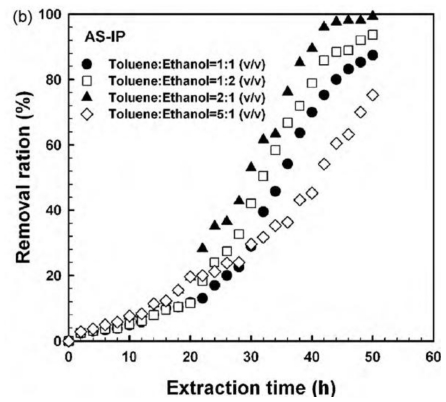
Removal ratio of aspirin from MIP particles (212 μm) as a function of the time of incubation with different toluene:ethanol mixtures. Reprinted from Byun *et al.* [39] with permission from Elsevier.

**Figure 6:**
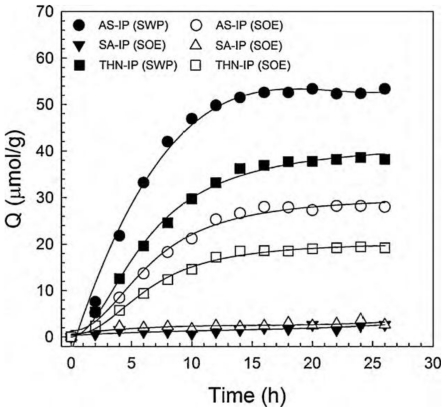
Rebinding profiles of aspirin (AS), salicylic acid (SA) or 1,2,3,4-tetrahydro-1-naphthol (THN) of the MIPs extracted templates using the Soxhlet extraction (SOE) or the swelling process (SWP). Reprinted from Byun *et al.* [39] with permission from Elsevier.

The nature of the solvent also plays an important role. Byun *et al.* [39] observed that aspirin could be removed from the imprinted networks described in Section 2.1 by swelling first in toluene and then adding ethanol to the medium. Faster and complete extraction of aspirin was achieved in toluene:ethanol 2:1 (v/v) under stirring (Figure 5). It is interesting to note that, although the use of Soxhlet extraction or swelling in toluene:ethanol medium led to almost complete template removal, the MIPs obtained behaved quite differently in subsequent experiments (Figure 6). In the rebinding tests, the MIPs extracted by swelling in toluene:ethanol medium reached the adsorption equilibrium faster and were able to capture almost twice the amounts taken by MIPs extracted with Soxhlet. The different performance can be explained by the fact that toluene is a good solvent for the polymer and thus the swelling in toluene:ethanol medium facilitates the removal of the template molecules from the inner regions of the MIP, making all cavities accessible to the solvent in a shorter time than the Soxhlet extraction with ethanol, a poor solvent of the MIP [39]. Changes in the imprinted cavities due to the different treatments cannot be discarded.

## 3. Physically-Assisted Extraction

This modality tries to optimize the extractive performance of the solvents, while minimizing solvent volume and operational time and costs.

### 3.1. Microwave-Assisted Extraction (MAE)

Microwaves (MW), electromagnetic waves in the 300–3,000,000 MHz range, directly interact with the molecules causing ionic conduction and dipole rotation [47,48]. Materials absorb MW energy, with the subsequent raise in temperature, depending on their dissipation factor (*tanδ; i.e.*, the ratio between the dielectric loss and the dielectric constant). Polar solvents and ionic solutions possess much larger *tan*δ than non-polar solvents [49].

MAE has been proposed to occur according to one of the following mechanism: (i) extraction into a solvent that absorbs the MW energy strongly; (ii) extraction into a mix of high and low *tan*δ solvents; and (iii) extraction into a non-absorbing solvent from a matrix with high *tan*δ [47]. Accordingly, two operational modalities have been developed [50]:

1. Pressurized MAE (PMAE), which uses closed MW-transparent vessels, filled with high *tanδ* solvents. The solvent absorbs the energy and the temperature raises, but boiling is prevented by the pressure inside the chamber.
2. Atmospheric MAE system, in open MW-transparent vessels, which is also named Focused Microwave-assisted Soxhlet extraction (FMASE) [51]. This technique employs low *tan*δ solvent and, thus, only the specimen to be extracted increases its temperature. Since the extraction is carried out under mild conditions, this modality is preferred for thermolabile substances.

In addition to the MW power, the selection of the solvent is a critical point because of its influence on the MW absorption, the solute solubility and the matrix solvation [49]. Differently from the improvement in conventional extraction observed as the volume of solvent increases, MAE yield may decrease due to inadequate stirring. MAE usually involves 10-fold less solvent (25–50 mL) than classical methods and requires much shorter times (3 min–1 h). These features lead to advantageous decrease in the costs associated to the solvents, energy and time, compared to classical extraction, and is considered to be a clean technique [31].

Regarding the application to MIPs, high MW power may make the template removal occur quite rapidly. However, excessive high temperature should be avoided in the case labile MIPs [10].Common particles size is in the 0.1–2 mm range; the smaller the particles, the greater the extraction yield. After MAE, the particles can be separated from the solvent by centrifugation or filtration [49]. For example, clenbuterol was removed from MIP particles (60 mg, 0.025–0.038 mm) using 20 mL of solvent (various solvents and mixtures were tested) in closed vessels that were exposed to MAE applying a 5 min heating ramp up to 100 °C. The experiments were carried out for 20 min to 4 h. Then, the systems was cooled, the solvent removed by filtration and the particles washed three times with 10 mL of methanol [10]. The highest extraction yield was achieved using strong organic acids, such as trifluoroacetic acid. However, an important weight loss was observed in the MIP particles probably due to hydrolysis. In the rebinding experiments, these treated MIPs exhibited an important deterioration in both binding affinity and selectivity, indicating the damage of the imprinted cavities. Therefore, a compromise between the reduction of bleeding during the analysis and the maintenance of the polymer integrity should be achieved. Mild acid conditions, as those provided by formic acid solutions, resulted to be adequate to achieve good removal yields without compromising the stability of the MIPs [10].

**Figure 7:**
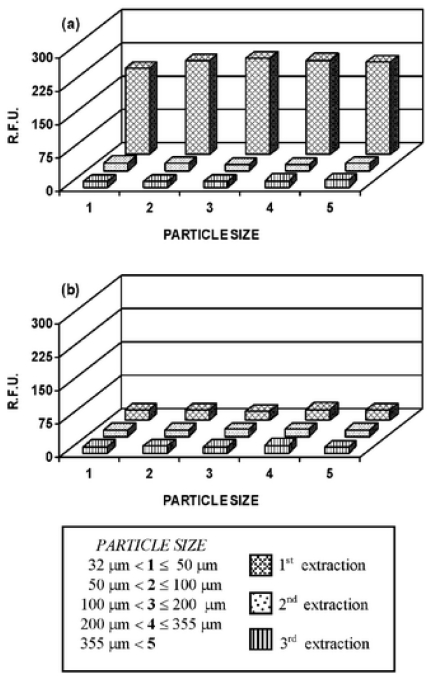
Fluorescence intensity of washing solutions containing estradiol extracted from imprinted (**a**) and non-imprinted (**b**) polymers having different size particles using microwave-assisted extraction (MAE). Reprinted from Bravo *et al.* [52] with permission from the Royal Society of Chemistry.

MAE has been also successfully applied for the removal of estradiol from MIPs prepared with methacrylic acid as functional monomer and ethyleneglycol dimethacrylate as cross-linker [52]. MIP particles (30 mg, 0.032–0.355 mm) in methanol containing 10%v/v acetic acid (20 mL) were placed in the vessels exposed to MAE over a 5 min heating ramp period up to 100 °C. This temperature was maintained for 20 min. Then, the solvent was removed by filtration and the MIP particles were extracted again under the same conditions. In total, three extraction cycles were performed for MIP particles of different sizes. Non-imprinted networks (NIPs) were similarly processed. Fluorimetric analysis of the solvent used in each extraction cycle indicated that complete estradiol removal was achieved in the first round (Figure 7). Compared to the most common extraction methods (incubation and Soxhlet), this procedure reduces the time required by several hours. In addition, less solvent is used [52].

### 3.2. Ultrasound-Assisted Extraction (UAE)

Ultrasound is a cyclic sound pressure with a frequency greater than 20 kHz. Ultrasonic energy causes an effect known as cavitation, which leads to the formation of small bubbles in liquid media and the mechanical erosion or rupture of solid particles. As a consequence, local increase in temperature (which favors solubility and diffusivity) and in pressure (which favors penetration and transport) increases [53,54]. Ultrasound radiation can be applied using suitable baths or probes; the latter enable a more localized treatment but the cost is high and the number of samples that can be processed each time is smaller [55]. Ultrasounds have been shown suitable to assist the polymerization step of MIPs, facilitating template solubilization and degassing [56–58]. In a typical ultrasound-assisted extraction (UAE) procedure, the polymer sample is placed in contact with a given volume (10–30 mL) of solvent and then ultrasounds are applied for 3–60 min. Several cycles, replacing the solvent, are usually carried out. There is also the possibility of establishing a continuous flow of the solvent through the chamber where the polymer is placed. After extraction, the polymer is recovered by centrifugation or filtration. Since the increase in temperature during ultrasound radiation is not too high, UAE is adequate for thermolabile templates that cannot withstand Soxhlet conditions.

UAE has been used to remove kaempferol from MIP microspheres prepared suing acrylamide or 2-vinylpyridine as functional monomers [59]. Complete template removal was achieved by placing the MIPs in acetic acid/methanol 1/9 v/v medium (50 mL) applying ultrasounds for 30 min. The process was repeated three times and the polymers were washed twice with methanol, also applying ultrasounds for 30 min, in order to eliminate acetic acid. The authors observed that more kaempferol was removed in the first UAE (1008 μg/g in 30 min) than using Soxhlet for 24 h (902 μg/g) [59]. Similarly, atrazine and indole-3-acetic acid have been removed from magnetic MIP beads by washing with 10% v/v acetic acid in methanol under ultrasonic agitation [60,61]. Ractopamine was removed from similarly prepared magnetic MIP beads by washing with methanol:water:acetic acid (85:5:10, v:v:v) under ultrasonic agitation [62].

MIP particles prepared using rutin (3,3′,4′,5,7-pentahydroxyflavone-3-(O-rhamnosylglucoside)) as template were subjected to an optimization study of the removal process using UAE and then the data were compared to those obtained with Soxhlet and MAE [63]. UAE was carried out in methanol:acetic acid (9:1, v/v) for various times. The removal yield increased with the solvent/MIP ratio, achieving a maximum at 40 mL/g, and the ultrasonication time, reaching an equilibrium after 1 h. Under those conditions, the amount of template removed by UAE was notably larger than that achieved applying the other techniques (Table 1).

### 3.3. Pressurized Liquid Extraction (PLE)

Pressurized fluid extraction, also called accelerated solvent extraction, equipments were commercially launched in 1995 [64]. The technique involves the use of heated organic and aqueous solvents in the liquid phase at pressure high enough to prevent boiling. The use of high temperature (50–200 °C) and pressure (10–14 MPa) greatly facilitate the penetration of the solvent into the matrix to be extracted (due to a decrease in viscosity and surface tension); the extraction requiring less solvent (15 mL for 10 g sample) and time (10–25 min) to be completed [65,66]. The high temperature facilitates the dissolution of the substance to be extracted and diminishes the interaction forces with the matrix [67,68].

PLE is commonly carried out by placing the sample (previously dried and with particle size below 2 mm) in a stainless steel chamber, which is then filled with the solvent. Static extraction at controlled temperature and pressure occurs for 5–10 min and then the solvent is delivered to a collector. The chamber is purged with nitrogen to remove all solvent and new solvent can be introduced in the chamber for another extraction cycle (1–2 cycles may be sufficient for certain applications) [67–69]. The extraction/purge cycles are intended to imitate the solvent cycles that occur in the Soxhlet apparatus, although in a more efficient way [27,70]. Nevertheless, PLE can be also modified to occur in continuous mode [71]. In any case, the main critical variables are temperature and time of extraction [72]. A relevant advantage of this technique is the versatility of solvents and mixtures that can be used for the extraction, making it suitable for the removal of almost any substance [69,73]. Furthermore, it can be easily automated for the simultaneous or sequential extraction of a relevant number of samples. However, it is even more expensive than MAE or the extraction with supercritical fluids (SFE) [74].

Since this technique is of relatively recent introduction, few examples of the application of PLE to the template removal are as yet available. Removal of enrofloxacin from MIPs applying PLE resulted to be as high as 99.9%, although detailed experimental conditions were not reported [75]. Tetracycline has also been successfully removed from imprinted xerogels with the use of methanol and 60 cycles of PLE at 70 °C and 105 bars, applying static extraction for 10 min per cycles. In this case, the removal also revealed that the template molecule had been converted to its epimer, 4-epitetracycline, and other molecules during the imprinting process [76].

## 4. Extraction with Supercritical or Subcritical Fluids

### 4.1. Supercritical Fluid Extraction (SPE)

Supercritical fluids exhibit features in between those of liquids (high solvation ability) and gases (high diffusivity), which is quite favorable for the diffusion into solid networks and for solubilizing a wide variety of substances. These features make supercritical fluids particularly attractive for extraction.

The main components of a SPE extractor are the source of the high purity supercritical fluid, a pump to deliver the fluid at constant pressure and flow, a thermostated extraction chamber, a valve to enable a controlled depressurization of the chamber, and a trap to collect the extracted substances. In commercial equipment, the supercritical fluid is pumped at a pressure above its critical point towards the extraction chamber. The extraction chamber has to be maintained at a temperature above the critical point. The extraction can be carried out in static (extraction cell filled with the fluid at equilibrium), dynamic (the supercritical fluid continuously flows through the cell) or recirculation (the same fluid is pumped through the chamber several times) modes. Then the supercritical fluid is propelled towards the trap [77]. Several interrelated factors should be adjusted to optimize the extraction from each material, such as the flow of the supercritical fluid, the pressure, the temperature, the extraction modes (which can be combined), the presence of a cosolvent, extraction time, and the system to collect the extracted substances [74,78].

The extraction ability of a supercritical fluid is very dependent on its density, which can be tuned with the pressure and the temperature. The greater the density, the better the interaction is with the solutes. An increase in pressure raises the density, but the diffusivity decreases. In general, the supercritical fluids have low surface tension values, which facilitate the penetration into polymer matrices, and also a low dielectric constant, which favors the solubilization of hydrophobic templates. Supercritical CO_2_ at 2 Kbar have ɛ values of 1.8, while supercritical water at the critical point exhibit ɛ values of 6, performing as non-polar solvents. Therefore, small, lipohilic solutes are the most prone to dissolve in supercritical fluids. The presence of polar groups in the template structure may notably decrease its solubility in the supercritical fluid [10].

Supercritical CO_2_ is the most used supercritical fluid due to its low critical parameters (32 °C, 73 atm), availability at high purity, innocuousness for humans and the environment, and non-flammability [27]. Although it can solubilize large lipohilic compounds and moderately polar substances, the extraction of polar solutes strongly attracted by a polymer matrix requires the addition of a cosolvent [31,71]. The cosolvent is usually an organic polar solvent (e.g., methanol, ethyl acetate, or acetonitrile) that is added in a low proportion to the supercritical fluid, resulting in an increase not only in the polarity but also in the density. In the case of template removal from MIPs, the addition of the cosolvent may have a double meaning: (i) to swell the polymer matrix, enhancing the diffusion; and (ii) to break the template-imprinted cavity interactions, displacing the template.

Examples of the use of supercritical CO_2_ for the template removal from MIPs are still few, probably because the equipment is not cheap and many parameters should be optimized for each system [74]. Clenbuterol, a polar compound, was removed from MIPs by placing the polymer particles into a stainless steel column fitted to a supercritical fluid chromatography equipment [10]. The mobile phase was supercritical CO_2_ containing 30% of methanol or methanol–acetic acid at various ratios. The temperature was set at 100 °C, the flow rate 1 ml min^−1^, the back pressure 200 bar, and the extraction time was varied between 1 and 18 h. The procedure enabled the removal of a large proportion of template, but no complete extraction was achieved. In the case of 2,4,6-trinitrotoluene-MIP porous beads, it has been reported that Soxhlet extraction followed by supercritical CO_2_ extraction under mild conditions (150 bar, 50 °C) leads to >99.7% template removed [79]. Furthermore the extracted beads maintained their integrity and porosity after the treatment. It has been recently shown that successive cycles of impregnation/extraction of templates in/from preformed materials using supercritical CO_2_ can render imprinted cavities, resulting in a post-imprinting effect [80].

### 4.2. Subcritical Water Extraction (SWE)

The suitability of water (the cheapest and greenish solvent) to remove templates can be notably improved when assisted by high pressure (10–60 bar) and temperature (100–374 °C) [81]. Extraction with subcritical water, also named as pressurized hot-water extraction (PHWE) or superheated-water extraction (SWE), was introduced in the mid-90s by Hawthorne’s group [82]. The interest of SWE is based on the large reduction in polarity that liquid water undergoes when heated at high temperature [83]. The decrease in dielectric constant from 80 at 25 °C to 27 at 250 °C and 50 bar and even to 10 at temperature above 300 °C opens the possibility of solubilizing a wide range of polar, ionic and non-polar compounds [71,84] The concomitant decrease in surface tension and viscosity markedly raises the diffusivity and mass transfer rate [85]. On the other hand, the thermal energy can break van der Waals forces, hydrogen bonds or dipole-dipole interactions among solute molecules as well as between the solute and the matrix. The high pressure forces water to penetrate into otherwise inaccessible regions [84]. Thermodynamic and kinetic models have been developed to explain the extraction mechanism behind SWE [86]. According to these models, the extraction involves desorption of the solute from the binding sites (diffusion-controlled step), followed by elution from the matrix due to partitioning of the solute into the solvent (matrix-solvent partition-controlled step).

The SWE unit is quite simple, includes a water reservoir, a medium-pressure pump, an oven, and a restrictor, and can be coupled on-line coupling with different techniques. Although SWE is often referred as a solvent-free technique, most of the time the aqueous extract is collected in an organic solvent. The main limitation of the technique, which can be carried out in static and dynamic modes, is that it is not suitable for labile templates and polymer matrices [74,87].

**Figure 8:**
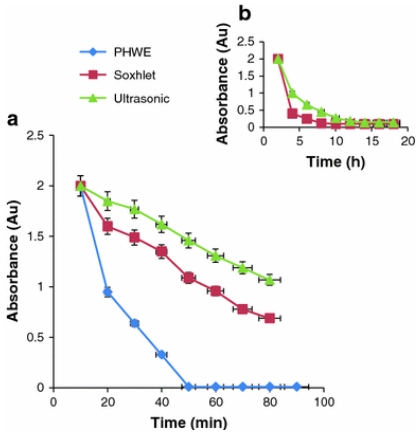
Absorbance of chlorophyll in each washing at: (**a**) 10 min intervals for the three extraction methods; and (**b**) at 2 h intervals for Soxhlet and ultrasound-assisted extraction. Reprinted from Batlokwa *et al.* [88] with permission from Springer.

A detailed report about the application of SWE to the removal of chlorophyll, quercetin and phthalocynine used as templates of MIPs has recently been published [88]. MIPs (800 mg) were extracted with water (34 mL, 2 mL/min) at 50 atm and 220 °C for chlorophyll and phthalocynine and 235 °C for quercitin. Complete template removal was achieved in 70 min. Furthermore, the obtained MIPs were able to rebind 99.6–100% template, which indicates that the imprinted cavities were not altered during the template removal. This report also compares the template removal by SWE with the data obtained applying Soxhlet and ultrasound-assisted extraction, which were carried out using up to nine times fresh 80 mL methanol aliquots at 70 °C for up to 16 h. The results (Figure 8) clearly evidenced the advantage of SWE in terms of both removal yield and time.

## 5. Conclusions and Future Trends

The information described above clearly indicates that the selection of an effective removal technique is not an easy task, mostly because of the tailored-nature of the MIP. Such an affinity and specificity for a given template is also responsible for making the removal process difficult, particularly when traditional techniques such as incubation or Soxhlet extraction are used. Nevertheless, several parameters may be tuned in order to improve the yield of template recovery, for example the amount and particle size of MIP, the nature and volume of solvent, and the operation time. Extraction assisted by ultrasounds (UAE), microwaves (MAE), or heating under pressure (PLE) has been shown to notably increase the template removal while using less solvents and time. However, drastic conditions may cause the MIP and the imprinted cavities to distort and, consequently, they become less efficient in rebinding and selectivity. Extraction with subcritical water (PHWE) or supercritical CO_2_ (SFE) has the advantage of combining environmental-friendly principles with high efficiency. Operational costs and, in some cases, stability-related problems may be the two main disadvantages of this technique. If a careful selection of the operational conditions is made, physically-assisted and subcritical/supercritical fluid extraction techniques may avoid the template removal to be the limiting step in the MIP preparation process, both from the point of view of time spent and of the working of the imprinted cavities. Advances in operation in continuous mode, automation of the template removal process, and on-line integration with instrumental techniques for real-time monitoring of the template extracted, are expected to notably optimize the cost/efficiency of the removal phase. In any case, it is essential to keep in mind that the objective of template removal is to make the cavities available for template rebinding, maintaining the conformation upon synthesis as close as possible.

## Figures and Tables

**Table 1:** Conditions and yield of the extraction processes tested for removal of rutin from MIPs. Reproduced from Peng *et al.* [63] with permission from the Royal Society of Chemistry.

Elution methods	Elution liquid-solid ratio (mL/g)	Elution time (h)	Elution capacity (Q, mg/g)

Soxhlet extraction	100	24	547
Ultrasonic extraction	40	1	815
Microwave-assisted extraction	40	5	326

a Q, elution capacity; Q = Ce × eluent volume [mL]/adsorbent mass [g], where Ce represents the concentration of analyte in the eluent.

## References

[1] Alvarez-Lorenzo C, Concheiro A (2004). Molecularly imprinted polymers for drug delivery. J. Chromatogr. B.

[2] Lasakova M, Jandera P (2009). Molecularly imprinted polymers and their application in solid phase extraction. J. Sep. Sci.

[3] Lieberzeit PA, Dickert FL (2009). Chemosensors in environmental monitoring: Challenges in ruggedness and selectivity. Anal. Bioanal. Chem.

[4] Bui BTS, Haupt K (2010). Molecularly imprinted polymers: Synthetic receptors in bioanalysis. Anal. Bioanal. Chem.

[5] Alvarez-Lorenzo C, Concheiro A, Arshady R, Kono K (2006). Molecularly imprinted gels and nano- and microparticles. Manufacture and applications. Smart Nano- and Microparticles.

[6] Poma A, Turner APF, Piletsky SA (2010). Advances in the manufacture of MIP nanoparticles. Trends Biotechnol.

[7] Hu Y, Li Y, Liu R, Tan W, Li G (2011). Magnetic molecularly imprinted polymer beads prepared by microwave heating for selective enrichment of β-agonists in pork and pig liver samples. Talanta.

[8] Cederfur J, Pei Y, Zihui M, Kempe M (2003). Synthesis and screening of a molecularly imprinted polymer library targeted for penicillin G. J. Comb. Chem.

[9] Nicholls IA, Andersson HS, Golker K, Henschel H, Karlsson BCG, Olsson GD, Rosengren AM, Shoravi S, Suriyanarayanan S, Wiklander JG (2011). Rational design of biomimetic molecularly imprinted materials: Theoretical and computational strategies for guiding nanoscale structured polymer development. Anal. Bioanal. Chem.

[10] Ellwanger A, Berggren C, Bayoudh S, Crecenzi C, Karlsson L, Owens PK, Ensing K, Cormack P, Sherrington D, Sellergren B (2001). Evaluation of methods aimed at complete removal of template from molecularly imprinted polymers. Analyst.

[11] Luliński P, Maciejewska D, Bamburowicz-Klimkowska M, Szutowski M (2007). Dopamine-imprinted polymers: Template-monomer interactions, analysis of template removal and application to solid phase extraction. Molecules.

[12] Batlokwa BM, Mokgadi J, Nyokong T, Torto N (2011). Optimal template removal from molecularly imprinted polymers by pressurized hot water extraction. Chromatographia.

[13] Shea KJ, Sasaki DY, Stoddard GJ (1989). Fluorescence probes for evaluating chain solvation in network polymers. An analysis of the solvatochromic shift of the dansyl probe in macroporous styrene-divinylbenzene and styrene-diisopropenylbenzene copolymers. Macromolecules.

[14] Ou SH, Wu MC, Chou TC, Liu CC (2004). Polyacrylamide gels with electrostatic functional groups for the molecular imprinting of lysozyme. Anal. Chim. Acta.

[15] Levi L, Srebnik S (2010). Simulation of protein-imprinted polymers. 1. Imprinted Pore properties. J. Phys. Chem. B.

[16] Lanza F, Sellergren B (2000). The application of molecular imprinting technology to solid phase extraction. Chromatographia.

[17] Szumski M, Buszewski B (2004). Molecularly imprinted polymers: A new tool for separation of steroid isomers. J. Sep. Sci.

[18] Martin P, Jones GR, Stringer F, Wilson ID (2003). Comparison of normal and reversed-phase solid phase extraction methods for extraction of β-blockers from plasma using molecularly imprinted polymers. Analyst.

[19] Yungerman I, Srebnik S (2006). Factors contributing to binding-site imperfections in imprinted polymers. Chem. Mater.

[20] Fu GQ, Yu H, Zhu J (2008). Imprinting effect of protein-imprinted polymers composed of chitosan and polyacrylamide: A re-examination. Biomaterials.

[21] Dirion B, Lanza F, Sellergren B, Chassaing C, Venn R, Berggren C (2002). Selective solid phase extraction of a drug lead compound using molecularly imprinted polymers prepared by the target analogue approach. Chromatographia.

[22] Boyd B, Björk H, Billing J, Shimelis O, Axelsson S, Leonora M, Yilmaz E (2007). Development of an improved method for trace analysis of chloramphenicol using molecularly imprinted polymers. J. Chromatogr. A.

[23] Schirmer C, Meisel H (2009). Chromatographic evaluation of polymers imprinted with analogs of chloramphenicol and application to selective solid-phase extraction. Anal. Bioanal. Chem.

[24] D’Oleo R, Alvarez-Lorenzo C, Sun G (2001). A new approach to design imprinted polymer gels without using a template. Macromolecules.

[25] Moritani T, Alvarez-Lorenzo C (2001). Conformational imprinting effect on stimuli-sensitive gels made with an “imprinter” monomer. Macromolecules.

[26] Suryanarayanan V, Wu CT, Ho KC (2010). Molecularly imprinted electrochemical sensors. Electroanalysis.

[27] Focant JF, Pirard C, de Pauw E (2004). Automated sample preparation-fractionation for the measurement of dioxins and related compounds in biological matrices: A review. Talanta.

[28] Raynie DE (2006). Modern extraction techniques. Anal. Chem.

[29] Hyötyläinen T (2009). Critical evaluation of sample pretreatment techniques. Anal. Bioanal. Chem.

[30] Kronholm J, Hartonen K, Riekkola ML (2007). Analytical extractions with water at elevated temperatures and pressures. Trends Anal. Chem.

[31] Tobiszewski M, Mechlińska A, Zygmunt B, Namieśnik J (2009). Green analytical chemistry in sample preparation for determination of trace organic pollutants. Trends Anal. Chem.

[32] Soxhlet F (1879). Die gewichtsanalytische Bestimmung des Milchfettes. Polytechnisches J. (Dingler’s).

[33] Luque de Castro MD, Priego-Capote F (2010). Soxhlet extraction: Past and present panacea. J. Chromatogr. A.

[34] Mullett WM, Lai EPC (1998). Determination of theophylline in serum by molecularly imprinted solid-phase extraction with pulsed elution. Anal. Chem.

[35] Turiel E, Martín-Esteban A, Tadeo JL (2007). Molecular imprinting-based separation methods for selective analysis of fluoroquinolones in soils. J. Chromatogr. A.

[36] Díaz-Alvarez M, Turiel E, Martín-Esteban A (2009). Selective sample preparation for the analysis of (fluoro)quinolones in baby food: Molecularly imprinted polymers versus anion-exchange resins. Anal. Bioanal. Chem.

[37] Turner NW, Holdsworth CI, Donne SW, McCluskey A, Bowyer MC (2010). Microwave induced MIP synthesis: Comparative analysis of thermal and microwave induced polymerisation of caffeine imprinted polymers. New J. Chem.

[38] Tunc Y, Hasirci N, Yesilada A, Ulubayram K (2006). Comonomer effects on binding performances and morphology of acrylate-based imprinted polymers. Polymer.

[39] Byun HS, Youn YN, Yun YH, Yoon SD (2010). Selective separation of aspirin using molecularly imprinted polymers. Sep. Purif. Technol.

[40] Hillberg AL, Brain KR, Allender CJ (2009). Design and evaluation of thin and flexible theophylline imprinted polymer membrane materials. J. Mol. Recognit.

[41] Alvarez-Lorenzo C, Hiratani H, Gómez-Amoza JL, Martínez-Pacheco R, Souto C, Concheiro A (2002). Soft contact lenses capable of sustained delivery of timolol. J. Pharm. Sci.

[42] Alvarez-Lorenzo C, Yañez F, Barreiro-Iglesias R, Concheiro A (2006). Imprinted soft contact lenses as norfloxacin delivery systems. J. Control. Release.

[43] Yanez F, Chauhan A, Concheiro A, Alvarez-Lorenzo C (2011). Timolol-imprinted soft contact lenses: Influence of the template/functional monomer ratio and the hydrogel thickness. J Appl Polym Sci.

[44] Bolisay LD, Culver JN, Kofinas P (2007). Optimization of virus imprinting methods to improve selectivity and reduce nonspecific binding. Biomacromolecules.

[45] Bolisay LD, Culver JN, Kofinas P (2006). Molecularly imprinted polymers for tobacco mosaic virus recognition. Biomaterials.

[46] Nemulenzi O, Mhaka B, Cukrowska E, Ramström O, Tute H, Chimuka L (2009). Potential of combining of liquid membranes and molecularly imprinted polymers in extraction of 17 β-estradiol from aqueous simples. J. Sep. Sci.

[47] Madej K (2009). Microwave-assisted and cloud-point extraction in determination of drugs and other bioactive compounds. Trends Anal. Chem.

[48] Cela R, Lorenzo RA, Casais MC (2002). Técnicas Analíticas de Separación en Química Analítica.

[49] Mandal V, Mohan Y, Hemalatha S (2007). Microwave assisted extraction—An innovative and promising extraction tool for medicinal plant research. Pharmacognosy Rev.

[50] Eskilsson CS, Björklund E (2000). Analytical-scale microwave-assisted extraction. J. Chromatogr. A.

[51] Luque-Garcia JL, Luque de Castro MD (2004). Focused microwave-assisted Soxhlet extraction: Devices and applications. Talanta.

[52] Bravo JC, Fernández P, Durand JS (2005). Flow injection fluorimetric determination of β-estradiol using a molecularly imprinted polymer. Analyst.

[53] Cintas P, Luche JL (1999). Green chemistry. The sonochemical approach. Green Chem.

[54] Luque-Garcia JL, Luque de Castro MD (2003). Ultrasound: A powerful tool for leaching. Trends Anal. Chem.

[55] Tadeo JL, Sánchez-Brunete C, Albero B, García-Valcárcel AI (2010). Application of ultrasound-assisted extraction to the determination of contaminants in food and soil samples. J. Chromatogr. A.

[56] Zheng X, Murray GM (1996). Synthesis and characterisation of site-selective ion-exchange resins templated for lead (II) ion. Sep. Sci. Tech.

[57] Jenkins AL, Uy MO, Murray GM (1999). Polymer based lanthanide luminescent sensors for detection of the hydrolysis product of the nerve agent Soman in water. Anal. Chem.

[58] Svenson J (2006). Ultrasound-assisted preparation of molecularly imprinted polymers: Effects on polymer morphology, binding, and chromatographic behavior. Anal. Lett.

[59] Zhu H, Wang Y, Yuan Y, Zeng H (2011). Development and characterization of molecularly imprinted polymer microspheres for the selective detection of kaempferol in traditional Chinese medicines. Anal. Methods.

[60] Hu Y, Liu R, Zhang Y, Li G (2009). Improvement of extraction capability of magnetic molecularly imprinted polymer beads in aqueous media via dual-phase solvent system. Talanta.

[61] Zhang Y, Li Y, Hu Y, Li G, Chen Y (2010). Preparation of magnetic indole-3-acetic acid imprinted polymer beads with 4-vinylpyridine and β-cyclodextrin as binary monomer via microwave heating initiated polymerization and their application to trace analysis of auxins in plant tissues. J. Chromatogr. A.

[62] Hu Y, Li Y, Liu R, Tan W, Li G (2011). Magnetic molecularly imprinted polymer beads prepared by microwave heating for selective enrichment of β-agonists in pork and pig liver samples. Talanta.

[63] Peng L, Wang Y, Zeng H, Yuan Y (2011). Molecularly imprinted polymer for solid-phase extraction of rutin in complicated traditional Chinese medicines. Analyst.

[64] Richter BE, Ezzell JL, Knowles DE, Hoefler F, Mattulat AKR, Scheutwinkel M, Waddell DS, Khurana TGV (1997). Extraction of polychlorinated dibenzo-p-dioxins and polychlorinated dibenzofurans from environmental samples using accelerated solvent extraction (ASE). Chemosphere.

[65] Richter BE (2000). Extraction of hydrocarbon contamination from soils using accelerated solvent extraction. J. Chromatogr. A.

[66] (2008). EPA Method 3545, Pressurised Fluid Extraction. Test Methods for Evaluating Solid Waste, Physical/Chemical Methods.

[67] Giergielewicz-Możajska H, Dąbrowski L, Namieśnik J (2001). Accelerated Solvent Extraction (ASE) in the analysis of environmental solid samples—some aspects of theory and practice. Crit. Rev. Anal. Chem.

[68] de Koning S, Janssen HG, Brinkman UAT (2009). Modern methods of sample preparation for GC analysis. Chromatographia.

[69] Nieto A, Borrull F, Pocurull E, Marce RM (2010). Pressurized liquid extraction: A useful technique to extract pharmaceuticals and personal-care products from sewage sludge. Trends Anal. Chem.

[70] Pronyk C, Mazza G (2009). Design and scale-up of pressurized fluid extractors for food and bioproducts. J. Food Eng.

[71] Mendiola JA, Herrero M, Cifuentes A, Ibañez E (2007). Use of compressed fluids for sample preparation: Food applications. J. Chromatogr. A.

[72] Ramos L, Kristenson EM, Brinkman UAT (2002). Current use of pressurised liquid extraction and subcritical water extraction in environmental analysis. J. Chromatogr. A.

[73] Carabias-Martínez R, Rodríguez-Gonzalo E, Revilla-Ruiz P, Hernández-Méndez J (2005). Pressurized liquid extraction in the analysis of food and biological samples. J. Chromatogr. A.

[74] Hyötyläinen T (2009). Critical evaluation of sample pretreatment techniques. Anal. Bioanal. Chem.

[75] Benito-Peña E, Martin S, Orellana G, Moreno-Bondi MC (2009). Water-compatible molecularly imprinted polymer for the selective recognition of fluoroquinolone antibiotics in biological samples. Anal. Bioanal. Chem.

[76] Mojica ERE, Autschbach J, Bright FV, Aga DS (2011). Tetracycline speciation during molecular imprinting in xerogels results in class-selective binding. Analyst.

[77] Fidalgo-Used N, Blanco-González E, Sanz-Medel A (2007). Sample handling strategies for the determination of persistent trace organic contaminants from biota samples. Anal. Chim. Acta.

[78] García-Rodríguez D, Carro-Díaz AM, Lorenzo-Ferreira RA (2008). Supercritical fluid extraction of polyhalogenated pollutants from aquaculture and marine environmental samples: A review. J. Sep. Sci.

[79] Bunte G, Hürttlen J, Pontius H, Hartlieb K, Krause H (2007). Gas phase detection of explosives such as 2,4,6-trinitrotoluene by molecularly imprinted polymers. Anal. Chim. Acta.

[80] Yañez F, Martikainen L, Braga ME, Alvarez-Lorenzo C, Concheiro A, Duarte CM, Gil MH, Sousa HC (2011). Supercritical fluid-assisted preparation of imprinted contact lenses for drug delivery. Acta Biomater.

[81] Herrero M, Cifuentes A, Ibanez E (2006). Sub- and supercritical fluid extraction of functional ingredients from different natural sources: Plants, food-by-products, algae and microalgae: A review. Food Chem.

[82] Hageman KJ, Mazeas L, Grabanski CB, Miller DJ, Hawthorne SB (1996). Coupled subcritical water extraction with solid-phase microextraction for determining semivolatile organics in environmental solids. Anal. Chem.

[83] Nerín C, Salafranca J, Aznar M, Batlle R (2009). Critical review on recent developments in solventless techniques for extraction of analytes. Anal. Bioanal. Chem.

[84] Teo CC, Tan SN, Hong Yong JW, Hew CS, Ong ES (2010). Pressurized hot water extraction (PHWE). J. Chromatogr. A.

[85] Ong ES, Cheong JSH, Goh D (2006). Pressurized hot water extraction of bioactive or marker compounds in botanicals and medicinal plant materials. J. Chromatogr. A.

[86] Kubatova A, Jansen B, Vaudiosot JF, Hawthorne SB (2002). Thermodynamic and kinetic models for the extraction of essential oil from savory and polycyclic aromatic hydrocarbons from soil with hot (subcritical) water and supercritical CO_2_. J. Chromatogr. A.

[87] Crescenzi C, Di Gorcia A, Nazzarri M, Samperi R (2000). Hot phosphate-buffered water extraction coupled on-line with liquid chromatography/mass spectrometry for analyzing contaminants in soil. Anal. Chem.

[88] Batlokwa BS, Mokgadi J, Nyokong T, Torto N (2011). Optimal template removal from molecularly imprinted polymers by pressurized hot water extraction. Chromatographia.

